# KIAA1429 promotes clear cell renal cell carcinoma progression by regulating MYC mRNA stability

**DOI:** 10.1016/j.isci.2026.115198

**Published:** 2026-03-03

**Authors:** Xu Zhu, Cheng Shen, Yong Zhang, Xinfeng Chen, Wei Zhang, Bing Zheng, Zhan Chen

**Affiliations:** 1 Department of Urology, Nantong First People’s Hospital, Nantong, China; 2 Institute of Urological Diseases, Department of Urology, Nantong First People’s Hospital, Nantong, China

**Keywords:** Health sciences, Biological sciences, Cancer systems biology, Cancer

## Abstract

KIAA1429, a core component of the m6A methyltransferase complex, is upregulated in clear cell renal cell carcinoma (ccRCC) and is associated with poor survival rates. Functional assays and *in vivo* models demonstrate that genetic depletion of KIAA1429 markedly inhibits tumor growth, as well as cancer cell proliferation, migration, and invasion. Mechanistically, KIAA1429 directly binds to the 3′ untranslated region (3′ UTR) of MYC mRNA and catalyzes site-specific m6A methylation, thereby enhancing transcript stability and augmenting MYC protein expression. Collectively, these findings identify KIAA1429 as a critical m6A-dependent post-transcriptional regulator of MYC and nominate the KIAA1429-MYC regulatory axis as a promising therapeutic target in ccRCC.

## Highlights

• KIAA1429 is upregulated in clear cell renal cell carcinoma and indicates poor prognosis
• Silencing KIAA1429 suppresses tumor growth, proliferation, migration, and invasion *in vitro*
• KIAA1429 promotes m6A modification and stabilizes MYC mRNA, enhancing its expression
• Targeting the KIAA1429-MYC axis presents a promising therapeutic strategy for ccRCC

## Graphical abstract

**Figure:**
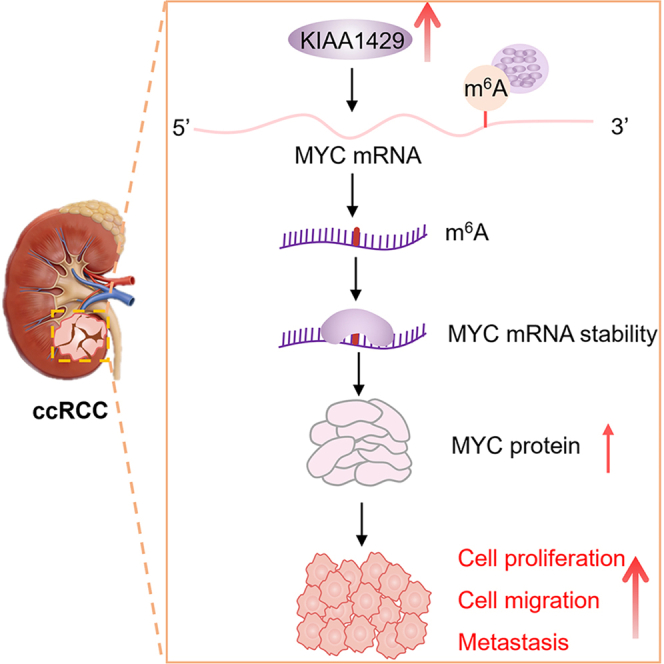

## Introduction

Renal cell carcinoma (RCC) ranks among the prevalent urologic cancers, constituting 2%–3% of all adult malignant neoplasms, with clear cell RCC (ccRCC) representing approximately 80% of all RCC cases.^1^^,^^2^ Despite its prevalence, RCC remains highly resistant to chemotherapy and radiotherapy, underscoring partial or radical nephrectomy as the primary surgical approach for localized RCC treatment.^3^^,^^4^ However, approximately 30%–50% of patients with RCC present with metastatic RCC (mRCC) at the time of diagnosis.^5^^,^^6^ Moreover, once renal cancer metastasizes, its clinical prognosis is significantly poor. Metastatic renal cancer exhibits a median survival of approximately 1 year, coupled with a 5-year survival rate below 10%.^7^^,^^8^ The pathogenesis of RCC is a complex mechanism, involving many genetic, epigenetic, and environmental factors.^9^ Accordingly, elucidating the molecular mechanisms that underlie RCC heterogeneity remains a major challenge. Furthermore, formulating a strategy is of vital importance for addressing this diagnostic and treatment challenge.

Recent studies have highlighted the critical role of N6-methyladenosine (m6A) RNA modification in cancer progression, including renal cell carcinoma.^10^ The dysregulation of m6A machinery components, such as writers, erasers, and readers, has been implicated in tumorigenesis and metastasis across various cancers.^11^ Importantly, identifying molecular targets and therapeutic strategies has become a central focus in urological oncology, as emerging evidence points to the clinical potential of targeting specific epigenetic and transcriptomic regulators in genitourinary cancers.^12^ KIAA1429, acknowledged as the largest known component of the m6A methyltransferase holocomplex, serves as a scaffold for the methyltransferase complex. It facilitates the binding between the catalytic core components of METTL3/METTL14/WTAP and the RNA substrate, thereby influencing the selective placement of m6A marks.^13^ Notably, KIAA1429 knockdown was reported to yield a 4-fold reduction in the median m6A peak score—exceeding the impact of METTL3 or METTL14 knockdown—underscoring the significant role of KIAA1429 with the methyltransferase complexes.^14^^,^^15^ Recent studies report that KIAA1429 is significantly upregulated in hepatocellular carcinoma tissues, correlating with the prognosis of patients with HCC. Moreover, KIAAA1429 mediates the m6A methylation of GATA3 pre-mRNA, which promotes the malignant phenotype of HCC cells.^16^ In breast cancer, studies have demonstrated that KIAA1429 regulates breast cancer proliferation through CDK1 in an m6A-independent manner.^17^ Similarly, in gastric cancer, studies report that KIAA1429 acts as an oncogene that promotes gastric cancer by regulating the stability of c-Jun mRNA.^18^ However, despite these insights and the emerging importance of m6A modifications in RCC,^19^ the precise function of KIAA1429 in ccRCC and its underlying regulatory mechanism remain largely unclear, highlighting a critical gap in knowledge.

In this study, we aimed to investigate the role and mechanism of KIAA1429 in ccRCC, focusing on its impact on the oncogene MYC through m6A-mediated mRNA regulation. By integrating bioinformatics analyses with extensive *in vitro* and *in vivo* functional experiments, we demonstrate for the first time that KIAA1429 is significantly upregulated in ccRCC and correlates with poor prognosis. Mechanistically, we reveal that KIAA1429 promotes ccRCC progression by enhancing the m6A modification and stability of MYC mRNA, thereby activating its oncogenic functions. These findings not only establish KIAA1429 as a prognostic biomarker and therapeutic target in ccRCC but also provide another insight into the epigenetic regulation of MYC via the m6A pathway in kidney cancer, addressing a previously unrecognized mechanism of ccRCC pathogenesis.

## Results

### Comprehensive analysis of the KIAA1429 in the TCGA and GEO database

**Figure 1:**
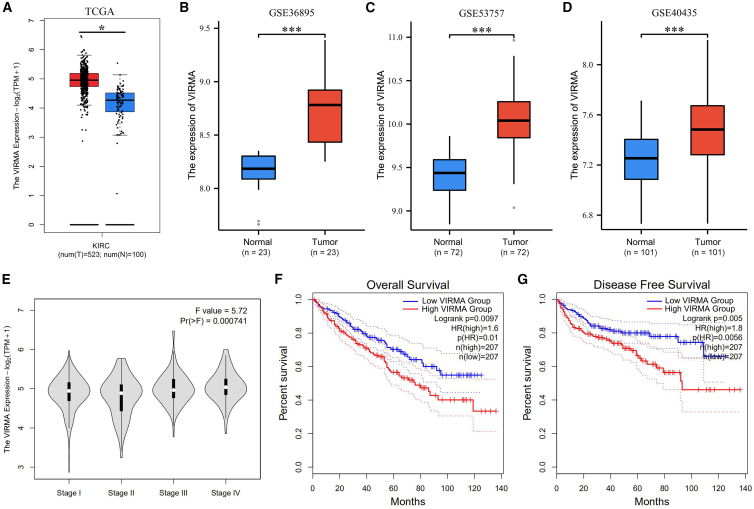
Comprehensive analysis of the KIAA1429 in the TCGA and GEO database (A) The expression of KIAA1429 in the TCGA-KIRCC database (tumor samples *n* = 523, normal samples *n* = 100). (B–D) The expression levels of KIAA1429 in ccRCC tissues and adjacent normal tissues (23 pairs of paired samples) in (B) GSE36895 dataset, (C) GSE53757 dataset (72 pairs of paired samples), and (D) GSE40435 dataset (101 pairs of paired samples). (E) The GEPIA database analyzed the expression of KIAA1429 in patients with ccRCC of different clinical stages. (F and G) The GEPIA database analyzed the effect of KIAA1429 mRNA expression on the OS and DFS of patients with ccRCC. Data are presented as the means ± SEM. ∗*p* < 0.05, ∗∗∗*p* < 0.001 by Student’s two-tailed *t* test.

The expression of KIAA1429 was analyzed in TCGA-KIRC using the GEPIA database, wherein the expression of KIAA1429 was observed to be upregulated in ccRCC tissues (Figure 1A). Furthermore, KIAA1429 expression was significantly higher in the ccRCC tissues from the GSE36895, GSE53757, and GSE40435 datasets (Figures 1B–1D). Further analysis of KIAA1429 expression in patients with ccRCC of different clinical stages revealed that the expression of KIAA1429 varied significantly among ccRCC patients at different clinical stages (Figure 1E).

In the ccRCC cohort, patients were stratified into the KIAA1429 high-expression group and KIAA1429 low-expression group based on the mean KIAA1429 value. Kaplan-Meier plotter survival analysis showcased the overall survival (OS) and disease-free survival (DFS) between these groups, signifying a poorer clinical prognosis associated with high KIAA1429 expression (OS: hazard ratio [HR] = 1.60, *p* = 0.0097; Figure 1F; DFS: HR = 1.80, *p* = 0.005; Figure 1G). Thus, these findings indicate the adverse impact of elevated KIAA1429 levels on ccRCC prognosis.

### KIAA1429 mRNA and protein expression was significantly upregulated in ccRCC tissues and cell lines

**Figure 2:**
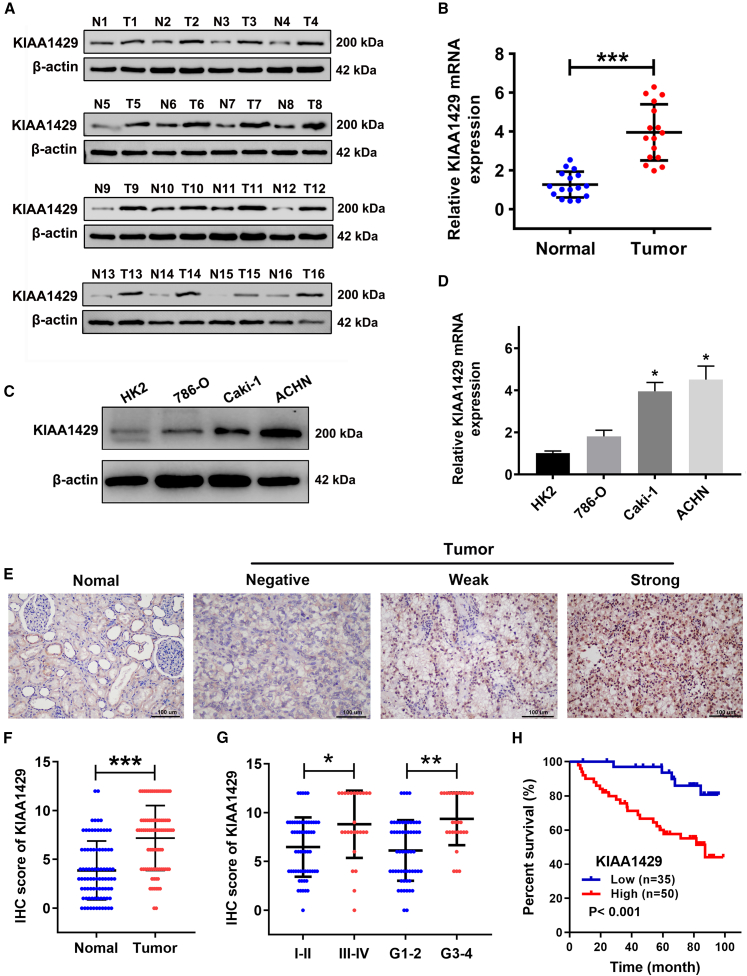
KIAA1429 mRNA and protein expression was significantly upregulated in ccRCC tissues and cell lines and its relationship to clinicopathological parameters (A and B) The protein and mRNA levels of KIAA1429 in ccRCC and normal kidney tissues. (C and D) KIAA1429 mRNA and protein expression in ccRCC cell lines and normal epithelium cell of renal tubule (HK2) cells. (E and F) The expression level of KIAA1429 in ccRCC tissue and surrounding normal renal tissue was detected using IHC. Scale bars, 100 μm. (G) The expression level of KIAA1429 in patients with ccRCC with different clinical stages and Fuhrman’s grade. (H) Effect of KIAA1429 expression on the overall survival of patients with ccRCC. T, tumor; N, normal. Data are presented as the means ± SEM. ∗*p* < 0.05, ∗∗*p* < 0.01, ∗∗∗*p* < 0.001 by Student’s two-tailed *t* test.

To corroborate the bioinformatics findings, we used western blot and quantitative reverse-transcription PCR (RT-qPCR) to analyze mRNA and protein levels of KIAA1429 in 16 pairs of fresh ccRCC tissues and normal kidney tissues. Western blotting confirmed that the protein levels of KIAA1429 were significantly upregulated in ccRCC tissues (Figure 2A), while RT-qPCR experiments also revealed a similar elevation in KIAA1429 mRNA levels (Figure 2B). Additionally, KIAA1429 expression was also detected in RCC cell lines using western blot and RT-qPCR experiments. KIAA1429 mRNA and protein levels were significantly high in the cells (Figures 2C and 2D).

### IHC analysis of KIAA1429 expression in ccRCC samples and its relationship to clinicopathological parameters

Immunohistochemical staining of cancer tissues from 85 patients with ccRCC was executed to explore the relationship between KIAA1429 expression and clinicopathological attributes. The analysis revealed that KIAA1429 was highly expressed in ccRCC tissues (Figures 2E and 2F) and the expression of KIAA1429 was significantly correlated with the survival status, TNM stage, tumor size, and Fuhrman grade (Figure 2G and Table 1). Additionally, Kaplan-Meier analysis showed that, compared to patients with ccRCC exhibiting low KIAA1429 expression, patients with high KIAA1429 expression had poorer clinical prognosis and poorer OS (*p* < 0.001; Figure 2H).

**Table 1:** Correlation between KIAA1429 expression and clinical case characteristics of ccRCC patients

Variable	No. of cases	KIAA1429 expression		*p* value
		Low	High	
Sample	85	35	50	
Age (year)				0.549
>60	39	17	21	
≤60	40	18	29	
Gender				0.825
Female	36	15	21	
Male	59	20	29	
T stage				0.007
T1 + T2	65	32	33	
T3 + T4	20	3	17	
Lymph node metastasis				0.479
Yes	78	33	45	
No	7	2	5	
Distant metastases				0.083
Yes	77	34	43	
No	8	1	7	
Clinical stage				0.001
I + II	59	31	28	
III + IV	26	4	22	
Grade				<0.001
G1 + G2	58	31	27	
G3 + G4	27	4	23	
Survival state				<0.001
Alive	56	30	26	
Dead	29	5	24	

Then, we included age, sex, clinical tumor grade, Fuhrman grade, lymph node status, metastasis status, and KIAA1429 expression level into the multivariate Cox regression model, wherein KIAA1429 expression was identified as an independent predictor of OS in patients with ccRCC (HR = 2.750, 95% confidence interval [CI] = 1.188–6.362, *p* = 0.032; Table 2). Collectively, these findings suggest that KIAA1429 may be involved in the progression of renal clear cell carcinoma and could serve as a prognostic marker.

**Table 2:** Univariate and multivariate Cox regression analysis of prognostic factors associated with ccRCC overall survival

Variable	Univariate analysis		Multivariate analysis	
	Hazard ratio (95% CI)	*p* value	Hazard ratio (95% CI)	*p* value
Age				
>60/≤60	1.238 (0.590–2.599)	0.572	Ref.	–
Gender				
Male/female	0.782 (0.421–1.453)	0.438	Ref.	–
T stage				
pT3–4/pT1–2	6.031 (3.282–9.551)	<0.001	3.643 (1.172–5.218)	0.038
Lymph node metastasis				
Yes/no	3.349 (1.261–8.893)	0.015	0.795 (0.266–2.378)	0.681
Distant metastasis				
Yes/no	5.492 (2.058–7.585)	<0.001	2.381 (0.731–7.760)	0.150
TNM stage				
III + IV/I + II	6.521 (2.204–10.145)	<0.001	7.210 (1.394–14.279)	0.018
Grade				
G_3_ + G_4_/G_1_ + G_2_	3.277 (1.789–6.002)	<0.001	3.088 (1.038–9.188)	0.043
KIAA1429 expression				
High/low	5.780 (1.997–16.730)	<0.001	2.750 (1.188–6.362)	0.032

### KIAA1429 promoted ccRCC cell proliferation, migration, and invasion *in vitro*

**Figure 3:**
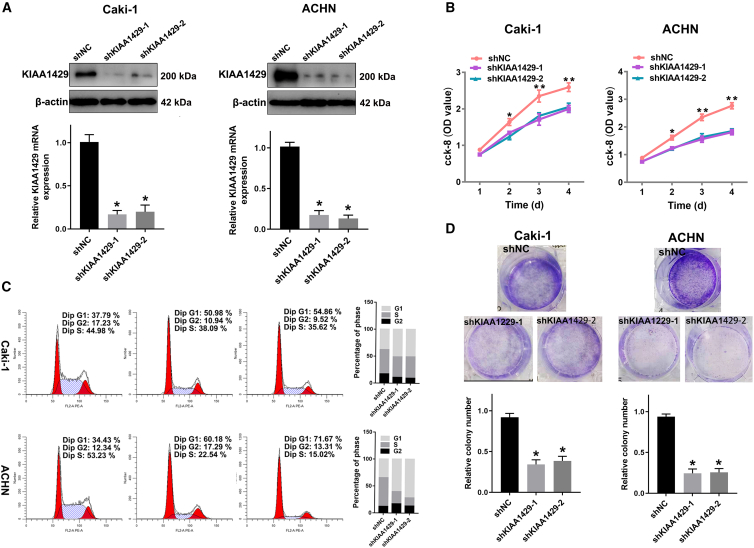
KIAA1429 promoted ccRCC cell proliferation *in vitro* (A) Knockdown efficiencies of KIAA1429 mRNA in ACHN and Caki-1 cells were detected using RT-qPCR; KIAA1429 protein expression decreased significantly in groups of shKIAA1429 in western blot analysis. (B and C) The effect of KIAA1429 knockdown on the proliferation of ccRCC cells was detected using a CCK-8 assay (B) and colony formation assay (C). (D) The effect of KIAA1429 on the cell cycle of ccRCC cells was detected using flow cytometry. Data are presented as the means ± SEM ∗*p* < 0.05, ∗∗*p* < 0.01 by Student’s two-tailed *t* test.

To further investigate the potential role of KIAA1429 in RCC cells, we constructed two lentiviral small hairpin RNAs (shRNAs) (LV-shKIAA1429-1/2) targeting KIAA1429 and a control lentiviral shRNA (LV-shNC). Caki-1 and ACHN cells were infected with LV-shKIAA1429 and LV-shNC, and stable cell lines were established through puromycin selection. Then, the knockdown efficiency of KIAA149 in the stably transfected cells was detected using RT-qPCR and western blot experiments (Figure 3A). The analyses revealed that LV-shKIAA1429 could significantly inhibit the expression of KIAA1429 protein and mRNA in Caki-1 and ACHN cells.

Furthermore, to explore the role of KIAA1429 in the proliferation of Caki-1 and ACHN cells, we performed cell colony formation and CCK-8 experiments. CCK-8 experiments revealed that the proliferation ability of Caki-1 and ACHN cells was significantly weakened after the knockdown of KIAA1429 (Figure 3B). Additionally, clonogenic experiments demonstrated that KIAA1429 knockdown resulted in a significant reduction in the number of clones in Caki-1 and ACHN cells (Figure 3D). Moreover, flow cytometry-based cell cycle analysis demonstrated an increase in the G1 phase and a decrease in the G2/M phase in KIAA1429-knockdown cells (Figure 3C). These results, thus, demonstrate that the knockdown of KIAA1429 could effectively inhibit the proliferation ability of Caki-1 and ACHN cells.

**Figure 4:**
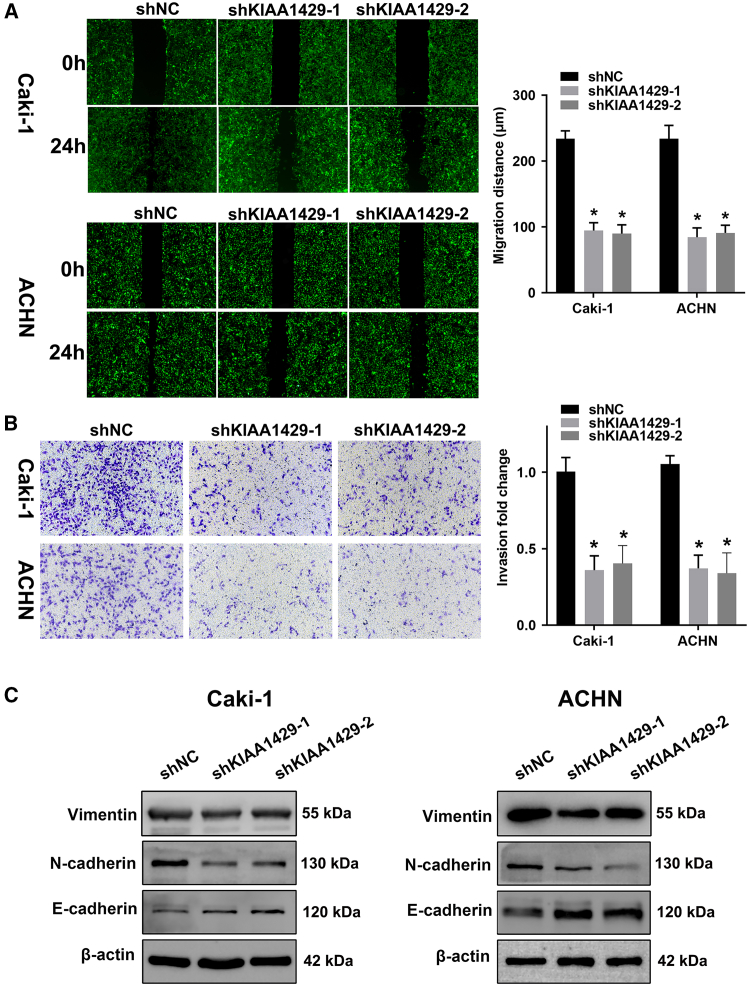
KIAA1429 promoted ccRCC cell migration and invasion *in vitro* (A and B) The effect of KIAA1429 knockdown on the migration and invasion of Caki-1 and ACHN cells were detected using a cell wound-healing assay (A) and transwell assay (B). (C) Western blot analysis of vimentin, N-cadherin, and E-cadherin protein expression in Caki-1 and ACHN cells with KIAA1429 knockdown. Data are presented as the means ± SEM. ∗*p* < 0.05 by Student’s two-tailed *t* test.

Migration and invasion assays, encompassing cell scratch experiments and transwell experiments, showed that, compared with control cells, the migration and invasion abilities were significantly inhibited in the KIAA1429 knockdown Caki-1 and ACHN cells, underscoring its contributory role in these processes (Figures 4A and 4B). Furthermore, the protein expression of vimentin, N-cadherin, and E-cadherin were detected by western blot. The result of western blot analysis showed that the expression of E-cadherin was up-regulated and those of vimentin and N-cadherin were down-regulated after inhibiting the expression of KIAA1429 in Caki-1 and ACHN cells (Figure 4C).

### KIAA1429 promoted tumorigenesis of ccRCC cells *in vivo*

**Figure 5:**
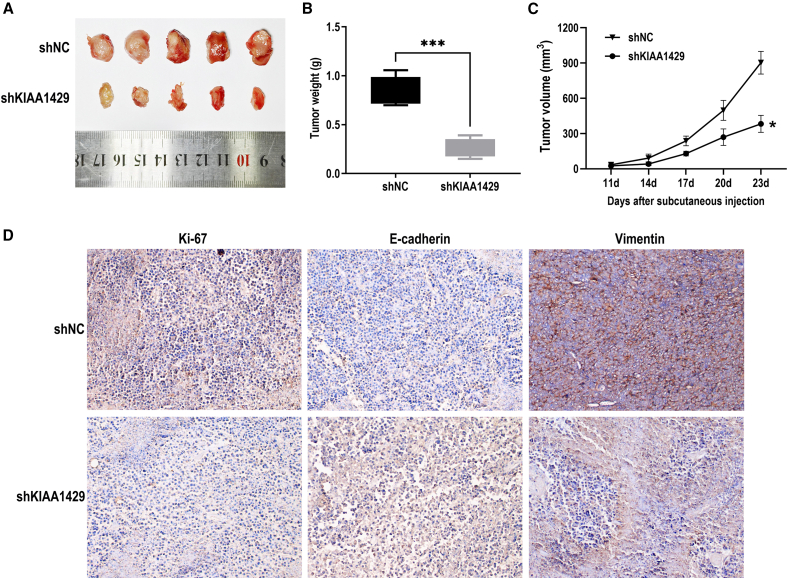
KIAA1429 promoted tumorigenesis of ccRCC cells *in vivo* (A) Subcutaneous tumor model of ACHN cells with KIAA1429 knockdown. KIAA1429 knockdown led to decreased tumor volume. (B and C) The changes in volume and weight were measured at the indicated weeks after mice were transplanted with the relevant cells. (D) IHC of vimentin, Ki-67, and E-cadherin protein expression in tumors with KIAA1429 knockdown. Data are presented as the means ± SEM. ∗*p* < 0.05, ∗∗∗*p* < 0.001 by Student’s two-tailed *t* test.

To further examine the effect of KIAA1429 knockdown on RCC cell growth, we conducted *in vivo* tumorigenesis experiments in nude mice subcutaneously xenografted. ACHN cells and KIAA1429-knockdown ACHN cells were injected subcutaneously into the axilla of female nude mice. The tumor growth curve experiments suggested that the growth of the transplanted tumors inoculated with KIAA1429-knockdown cells was significantly slower than that inoculated with control cells (Figure 5A). Moreover, the tumor growth rate of the KIAA1429 knockdown group was significantly inhibited (Figure 5C). Upon experiment conclusion, the tumors in the nude mice were isolated and weighed, revealing significantly reduced tumor weight in the KIAA1429-knockdown group compared to the control group (Figure 5B). Immunohistochemical analysis of tumor tissues revealed distinct expression patterns for Ki-67, vimentin, and E-cadherin. Compared with the KIAA1429 knockdown group, the control group exhibited stronger staining intensities for Ki-67 and vimentin, whereas E-cadherin expression was notably reduced (Figure 5D). Collectively, these results suggest that KIAA1429 knockdown inhibits ccRCC cell xenograft tumor growth.

### MYC might be a potential target for regulation by KIAA1429 in ccRCC

**Figure 6:**
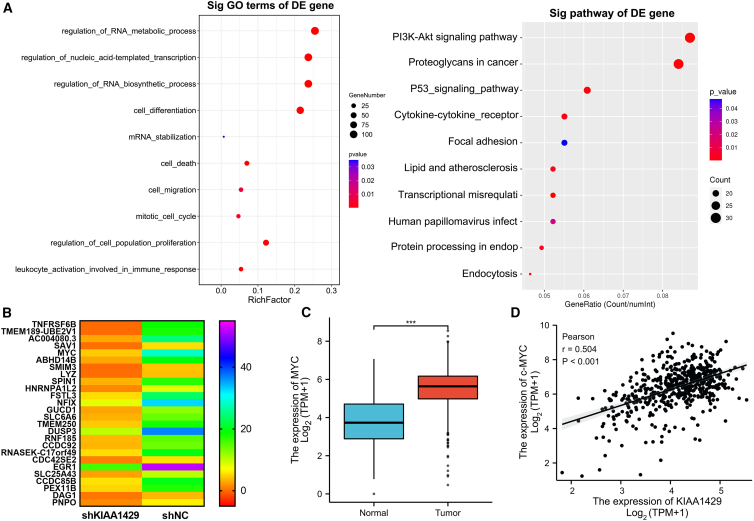
MYC might be a potential target for regulation by KIAA1429 in ccRCC (A) KIAA1429 has a significant effect on most RNA-specific processes, cell cycle, cell death, apoptosis, and intracellular signaling pathways, as revealed by GO and KEGG analyses. (B) Heatmap illustrating the downregulated genes in ACHN cells after KIAA1429 knockdown. (C) The expression level of MYC in ccRCC, obtained from the TCGA database. (D) Correlation analysis between the expression of MYC and KIAA1429. Data are presented as the means ± SEM. ∗∗∗*p* < 0.001 by Student’s two-tailed *t* test.

To gain insight into the downstream targets of KIAA1429 in ccRCC, we performed whole-transcriptome RNA sequencing (RNA-seq) to elucidate the regulatory significance of KIAA1429 in terms of genome-wide gene expression. Gene Ontology (GO) enrichment analysis revealed that the enriched gene sets were involved in most RNA-specific processes (RNA catabolic processes, RNA metabolic processes, RNA trafficking, localization, and translation), cell cycle, cell death, apoptosis, and intracellular signaling pathways (Figure 6A). Moreover, Kyoto Encyclopedia of Genes and Genomes (KEGG) signaling pathway revealed that P53 signaling pathway, chemokines, extracellular matrix, and PI3K-Akt signaling pathway were influenced by the downregulation of KIAA1429 (Figure 6A). Notably, RNA-seq results confirmed the role of KIAA1429 in promoting the progression of renal clear cell carcinoma. Previous studies report that KIAA1429 can directly bind to target gene mRNA. Related studies have also determined the potential target mRNA of KIAA1429 in HeLa cells using RIP-seq.^13^ Combined with our RNA-seq results, the MYC gene was selected as a possible downstream target of KIAA1429 in ccRCC, which was verified by RT-qPCR (Figure 6B).

**Figure 7:**
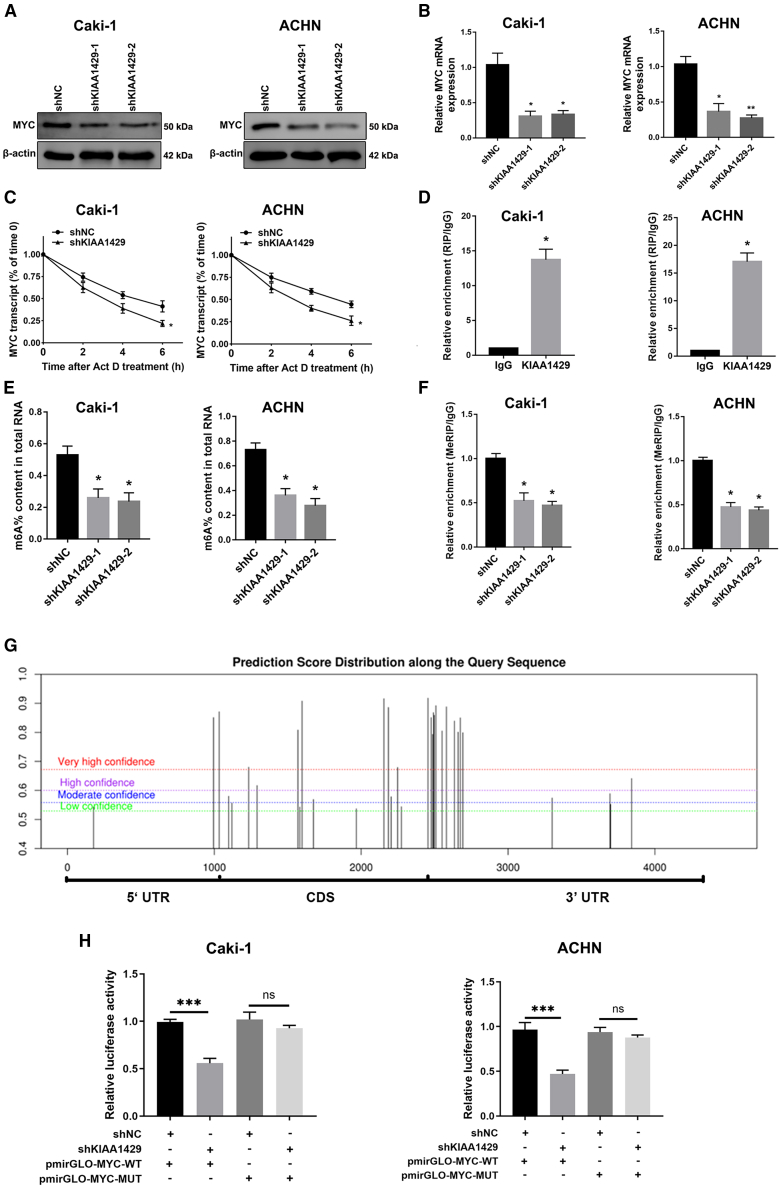
KIAA1429 regulated the expression of MYC mRNA and its stability in an m6A-dependent manner (A and C) KIAA1429 knockdown inhibits the protein and mRNA expression of MYC in ccRCC cells. (B) KIAA1429 knockdown decreased the stability of MYC mRNA. (D) RIP assay showed that KIAA1429 interacts with MYC mRNA in Caki-1 and ACHN cells. (E) KIAA1429 knockdown reduced overall m6A levels in Caki-1 and ACHN cells. (F) KIAA1429 knockdown reduced m6A levels in MYC. (G) m6A methylation sites on MYC were predicted using the SRAMP database. (H) The pmirGLO-MYC-WT or pmirGLO-MYC-MUT luciferase reporter was transfected into KIAA1429-knockdown and control Caki-1 and ACHN cells, and relative luciferase activity was measured. Data are presented as the ratio of firefly to Renilla luciferase activity. Data are presented as the means ± SEM. ∗*p* < 0.05, ∗∗*p* < 0.01, ∗∗∗*p* < 0.001 by Student’s two-tailed *t* test.

To elucidate KIAA1429-mediated molecular functions in RCC cells, we performed bioinformatic analysis to explore potential mRNAs regulated by KIAA1429. Analysis of the TCGA database revealed that MYC was upregulated in ccRCC (Figure 6C) and positively correlated with KIAA1429 expression (Figure 6D). To further verify the effect of KIAA1429 on the MYC gene, we examined the effect of knocking down KIAA1429 on the expression level of MYC in Caki-1 and ACHN cells using RT-qPCR and western blot experiments. Figures 7A and 7B show the protein and mRNA expression levels after knockdown of MYC.

### KIAA1429 regulated the expression of MYC mRNA and its stability in an m6A-dependent manner

KIAA1429, a main component in the m6A methyltransferase whole complex, primarily functions to catalyze the m6A modification of adenylate on mRNA. To elucidate the mechanism by which KIAA1429 influences MYC expression, KIAA1429-knockdown cells were treated with actinomycin D. The knockdown of KIAA1429 significantly decreased the stability of MYC mRNA (Figure 7C), suggesting that KIAA1429 increases the stability of MYC mRNA. Additionally, the RIP experiments revealed that KIAA1429 could significantly enrich MYC mRNA (Figure 7D). Finally, we investigated whether the global modification level of m6A in RCC cells was altered after KIAA1429 reduction. Notably, in KIAA1429-inhibited cells, a significant decrease in m6A levels was observed (Figure 7E). MeRIP experiments also demonstrated that KIAA1429 knockdown significantly decreased the m6A levels of MYC mRNA in ccRCC cells (Figure 7F). Subsequently, we employed the SRAMP database to predict m6A methylation sites on MYC, which were primarily concentrated near the 3′ UTR and stop codon (Figure 7G). Given prior evidence that KIAA1429 mediates m6A methylation preferentially in these regions,^10^ we constructed a pmirGLO-MYC-WT luciferase reporter containing the wild-type MYC 3′ UTR. As a control, we generated a mutant reporter (pmirGLO-MYC-MUT) by substituting adenine (A) with cytosine (C) at the predicted m6A site, based on previously identified methylation motifs.^13^ Using a dual-luciferase reporter assay, we found that knockdown of KIAA1429 significantly reduced luciferase activity from the wild-type reporter, but did not significantly affect the mutant reporter (Figure 7H). Taken together, KIAA1429 can be speculated to promote the m6A methylation modification of MYC mRNA.

## Discussion

The development of RCC is frequently associated with mutations in the VHL gene, leading to inactivation of the VHL protein and the accumulation of hypoxia-inducible factors (HIF-1α and HIF-2α).^20^ This creates a favorable microenvironment for tumor growth by promoting angiogenesis.^21^ Anti-angiogenic drugs, such as sorafenib and sunitinib, were developed to inhibit tumor angiogenesis based on this mechanism.^22^ However, many RCC patients exhibit only a brief response to these targeted therapies and eventually develop resistance.^23^ Given these challenges, it is imperative to explore mechanisms underlying RCC progression and to identify therapeutic targets. In this study, we identified aberrantly high expression of KIAA1429 in ccRCC through bioinformatics analysis and *in vitro* experiments, establishing its diagnostic and prognostic significance.

Research on chemical modifications has primarily focused on DNA and proteins, particularly on processes such as methylation, phosphorylation, and acetylation.^24^^,^^25^^,^^26^ In contrast, RNA undergoes over 100 distinct chemical modifications during synthesis.^27^ Despite their prevalence, the intricate processes, functions, and mechanisms underlying these modifications remain largely unexplored. Among these, m6A is recognized as the most abundant modification in eukaryotic RNA.^28^ The pathological significance of m6A dysregulation, particularly in cancer, has been the focus of numerous studies.^29^^,^^30^ Among the m6A methyltransferases, METTL3, METTL14, and WTAP have been extensively studied for their roles and mechanisms in various malignancies.^28^^,^^31^^,^^32^^,^^33^ For instance, KIAA1429 has been shown to promote liver cancer cell migration and invasion by enhancing m6A modification of ID2 mRNA, thereby suppressing ID2 expression.^34^ However, the specific functions and regulatory mechanisms of KIAA1429 in ccRCC remain unclear. Analysis of the TCGA and GEO database revealed that KIAA1429 expression is upregulated in ccRCC tissues, a finding further corroborated by immunohistochemistry (IHC) analysis. KIAA1429 expression was significantly associated with survival status, TNM stage, tumor size, and Fuhrman grade. Multivariate Cox regression analysis confirmed that elevated KIAA1429 expression is linked to poorer clinical outcomes, suggesting its potential as an independent prognostic marker for overall survival in ccRCC patients. Collectively, these findings underscore the therapeutic potential of targeting KIAA1429 in ccRCC.

Extensive studies have associated the oncogenic and tumor-suppressive roles of various m6A regulators with the development and progression of renal cell carcinoma.^35^ Studies have shown that ALKBH5 is highly expressed in renal cell carcinoma and may promote tumorigenesis by stabilizing AURKB mRNA through an m6A-dependent mechanism.^36^ Knockdown of METTL3 in RCC cells can promote the m6A-dependent translation of ABCD1 mRNA, which encodes the ATP-binding cassette subfamily D member 1 (ABCD1), effectively reducing cell migration and tumor spheroid formation.^37^ Our findings demonstrated that overall m6A levels were significantly reduced in KIAA1429-inhibited cells, highlighting the critical role of KIAA1429 in m6A modification. Further investigation into the function of KIAA1429 in RCC cell proliferation, through CCK-8 and colony formation assays, revealed that KIAA1429 knockdown significantly suppressed cell proliferation and induced G1 phase cell-cycle arrest. Additionally, subcutaneous xenograft tumorigenesis experiments in nude mice showed a marked reduction in tumor growth rates in the KIAA1429 knockdown group compared to controls. Collectively, these results indicate that KIAA1429 acts as an oncogene and plays a pivotal role in the progression of RCC.

To elucidate the molecular mechanisms by which KIAA1429 contributes to the pathogenesis of ccRCC, we conducted RNA-seq transcriptome analysis to identify potential target mRNAs influenced by KIAA1429. Our findings revealed that KIAA1429 downregulation affected several cancer-related signaling pathways, including the P53 signaling pathway, chemokines, extracellular matrix organization, and the PI3K-Akt signaling pathway. By integrating RNA-seq data with RT-qPCR validation, we identified MYC as a potential downstream target of KIAA1429 in ccRCC. Consistently, KIAA1429 knockdown led to a significant reduction in MYC expression, and a positive correlation between KIAA1429 and MYC expression was observed in RCC patient tissues. These results indicate that MYC plays a central role as a downstream target of KIAA1429 in RCC.

MYC, a well-established oncogene, is a key driver of cell proliferation and plays a pivotal role in various human cancers.^38^ Overactivation of MYC has been associated with RCC progression, including an increased risk of lymph node involvement and distant metastasis, establishing it as an independent prognostic factor.^39^ KIAA1429, an m6A methyltransferase, regulates the expression of target genes in an m6A-dependent manner. In KIAA1429-knockdown cells treated with actinomycin D, we observed a marked reduction in the stability of MYC mRNA. Furthermore, RIP and MeRIP assays confirmed that KIAA1429 enhances the enrichment of MYC mRNA and stabilizes it by increasing m6A levels. Dual-luciferase reporter assays showed that KIAA1429 directly binds the 3′ UTR of MYC mRNA and catalyzes site-specific m6A methylation. These findings collectively indicate that KIAA1429 may promote cell proliferation by regulating MYC expression in an m6A-dependent manner.

In conclusion, this study highlights the potential of KIAA1429 as an oncogene in ccRCC, attributed to its role in stabilizing MYC mRNA through the promotion of m6A methylation modifications. Moreover, we also underscore the significance of KIAA1429 in potentially targeting the MYC signaling pathway. This finding holds promise for the development of treatment strategies for ccRCC.

### Limitations of the study

However, our study has certain limitations. Due to a limited number of clinical samples, we analyzed KIAA1429 expression in only 85 pairs of renal cancer tissues. In future research, we aim to collect more patient samples, accumulate clinical data, and validate the clinical significance of KIAA1429 in a larger cohort. Although this study established a correlation between KIAA1429 and MYC expression, it may not fully elucidate the mechanisms by which KIAA1429 influences ccRCC progression. The effect of m6A modification on mRNA stability is mediated by specific reader proteins. To address this, we plan to investigate the reader proteins involved in the regulation of MYC mRNA by KIAA1429. Such studies will provide further evidence supporting the role of KIAA1429 in regulating MYC mRNA in an m6A-dependent manner.

## Resource availability

### Lead contact

Further information and requests for resources should be directed to and will be fulfilled by the lead contact, Zhan Chen (871427347@qq.com).

### Materials availability

All unique/stable reagents generated in this study are available from the lead contact with a completed materials transfer agreement.

### Data and code availability

All data in this paper will be shared by the lead contact upon request.

The datasets generated and/or analyzed during the current study have been deposited at the GEO and TCGA database repository and are publicly available as of the date of publication. Accession numbers are listed in the key resources table.

Any additional information required to reanalyze the data reported in this paper is available from the lead contact upon request.

## Acknowledgments

This work was supported by the 10.13039/501100005054Nantong University Special Research Fund for Clinical Medicine (grant no. 2024JQ009), Youth Project of Health Commission of Nantong City (QN2022017), Nantong Science and Technology Bureau (MS22019009), and Basic Research and Social Minsheng Plan Project (JC12022008). Thanks to The Cancer Genome Atlas Program for providing a wealth of analyzable public data.

## Author contributions

B.Z. designed this study. X.Z., Z.C., C.S., Y.Z., and W.Z. performed all the *in vitro* and *in vivo* experiments. Z.C., X.C., X.F.C. operated the data analysis in this study. Z.C. and C.S. produced this manuscript, which was examined and revised by B.Z. All authors have approved the submission of this manuscript. Z.C. and B.Z. confirm the authenticity of all the raw data.

## Declaration of interests

The authors declare no competing interests.

## STAR★Methods

### Key resources table

**Table:** 

REAGENT or RESOURCE	SOURCE	IDENTIFIER
**Antibodies**		

KIAA1429 Rabbit Polyclonal antibody	Proteintech	Cat#24761-1-AP
β-actin Rabbit Polyclonal antibody	Proteintech	Cat#66009-1-Ig
Vimentin Rabbit Polyclonal antibody	Proteintech	Cat#10366-1-AP
N-cadherin Rabbit Polyclonal antibody	Proteintech	Cat#66219-1-Ig
E-cadherin Rabbit Polyclonal antibody	Proteintech	Cat#20874-1-AP
MYC Rabbit Polyclonal antibody	Proteintech	Cat#10828-1-AP
HRP Goat Anti-Rabbit IgG	Proteintech	Cat#SA00001-2
HRP Goat Anti-Mouse IgG	Proteintech	Cat#SA00001-1
Ki-67 Polyclonal antibody	Proteintech	Cat#27309-1-AP

**Biological samples**		

Human tumor samples	This paper	–

**Chemicals, peptides, and recombinant proteins**		

Puromycin	Beyotime Biotechnology	Cat#ST551
Actinomycin D	MedChemExpress	Cat#HY-17559
Propidium Iodide	BD Biosciences	Cat#556463
TRIzol Reagent	Invitrogen	Cat#15596026
Protein A/G sepharose beads	Santa Cruz Biotechnology	Cat#sc-2003

**Critical commercial assays**		

CCK-8 reagent	Dojindo Molecular Technologies	Cat#CK04
PrimeScript™ RT Reagent Kit	Takara Bio	Cat#RR037A
SYBR Premix Ex Taq™ kit	Takara Bio	Cat#DRR081A
EpiQuik m6A RNA Methylation Quantification Kit	Merck Millipore	Cat#P-9005
Magna MeRIP™ m6A Kit	Merck Millipore	Cat#17-10499
Magna RIP™ RNA-Binding Protein Immunoprecipitation Ki	Millipore	Cat#17-700
Dual-Glo Luciferase Assay System	Promega	Cat#E2920

**Deposited data**		

GEO database (GSE36895, GSE53757, GSE40435)	NCBI GEO	https://www.ncbi.nlm.nih.gov/geo/
The Cancer Genome Atlas (TCGA) transcriptomic data	NCI Genomic Data Commons	https://portal.gdc.cancer.gov/
ACHN cell RNA-seq data	This paper (Deposited on GEO)	GEO: GSE276431; https://www.ncbi.nlm.nih.gov/geo/

**Experimental models: Cell lines**		

HK2 (human renal tubular epithelial cells)	ATCC	RRID: CVCL_0302
786-O	ATCC	RRID: CVCL_1051
ACHN	ATCC	RRID: CVCL_1067
Caki-1	ATCC	RRID: CVCL_0234

**Experimental models: Organisms/strains**		

BALB/c-nu mice, female, 4-6 weeks	Animal Experiment Center of Nantong University	–

**Oligonucleotides**		

shKIAA1429-1: AGGAGTGATCAGTGGATTATT	This paper	–
shKIAA1429-2: CCAAGAAATAGTTCGCTCTTA	This paper	–
shNC: TTCTCCGAACGTGTCACGT	This paper	–
Primer: KIAA1429-F: CCCAACGATGGCACGAATTAC	This paper	–
Primer: KIAA1429-R: GGGTGAGCACTTGTTACTGGA	This paper	–
Primer: MYC-F: CATCAGCACAACTACGCAGC	This paper	–
Primer: MYC-R: GCTGGTGCATTTTCGGTTGT	This paper	–
Primer: GAPDH-F: ACTTGGTATCGTGGAAGGACTC	This paper	–
Primer: GAPDH-R: GTAGAGGCAGGGATGATGTTCTG	This paper	–

**Software and algorithms**		

GraphPad Prism 8.02	GraphPad Software	–
SPSS	IBM	–
ImageJ	NIH	RRID:SCR_003070
R (version 4.2.1)	R Project	RRID: SCR_001905
Cell Quest Modfit software	Becton Dickinson	–

### Experimental model and study participant details

#### Patients and specimens

Human renal tissue samples were obtained from patients diagnosed with clear cell renal cell carcinoma (ccRCC) who underwent surgical resection at Nantong First People's Hospital. A total of 85 pairs of formalin-fixed paraffin-embedded (FFPE) ccRCC and matched adjacent normal kidney tissues were collected for immunohistochemical (IHC) analysis. Additionally, 16 pairs of fresh frozen ccRCC and adjacent normal tissues were obtained for Western blot and quantitative real-time PCR (qRT-PCR) validation. All patients were of Chinese ethnicity, with ages ranging from 38 to 79 years. The cohort included 58 males (68.2%) and 27 females (31.8%). Clinicopathological characteristics including TNM stage, Fuhrman grade, tumor size, lymph node status, and metastasis status were recorded for each patient.The detailed personal information of each participant can be found in Data S1. All participants provided written informed consent prior to sample collection. The study protocol was reviewed and approved by the Ethics Committee of the Second Affiliated Hospital of Nantong University (approval no. 2021KYS004) and conducted in accordance with the Declaration of Helsinki and the Ethical Guidelines for Medical and Health Research Involving Human Subjects. Patient samples were anonymized prior to analysis to protect confidentiality. The gender of the patients did not have a significant influence on the study.

#### Animal studies

4-6 week old female BALB/c nude mice were purchased from the Animal Experiment Center of Nantong University. Animals were housed in specific pathogen-free (SPF) conditions with controlled temperature (22-24°C), humidity (50-60%), and a 12-hour light/dark cycle. Mice were group-housed (5 per cage) in individually ventilated cages with sterile bedding and provided with autoclaved food and water *ad libitum*. KIAA1429 knockdown ACHN cells and control group cells were collected at a cell concentration of 2∗10^7^/ml and injected into the axilla of nude mice subcutaneously at 100ul. These mice were randomly divided into two groups, there were 5 mice in each group (n = 5). After the tumor transplantation model was established, when the tumor volume of the two groups was about 100 mm3, the *in vivo* tumor was recorded. The weight and tumor volume are measured twice each week. Tumor volume was calculated using the formula: tumor volume (mm^3^) = length × width2 × 0.52. After four weeks, the mice were euthanized by CO2 inhalation, and the tumors were excised and weighed. Once the diameter of the xenograft reaches 20 millimeters, or the volume reaches 2000 cubic millimeters, or the weight drops by 20%, the mouse is considered dead and the survival time is recorded. The animal studies were approved by the Animal Ethics Committee of Nantong University and conducted in accordance with the National Institutes of Health guide for the care and use of laboratory animals (approval no. S20220227-041).

#### Cell culture and transfection

The human normal renal tubular epithelial cells (HK2) and renal carcinoma cell lines (786-O, ACHN, Caki-1) were obtained from the American Type Culture Collection (ATCC). ATCC utilizes Promega PowerPlex® 21 System (for human cell lines) and confirms species via COI analysis. The receiving laboratory conducts an external inspection to confirm the growth characteristics of the cell line. Cells were tested for mycoplasma contamination upon receipt and before experimental use using the MycoAlert™ PLUS Mycoplasma Detection Kit (Lonza, Cat#LT07-705). All tests were negative. Routine testing was performed every 3 months during culture, to ensure that the cell line is free of mycoplasma. HK2 cells were cultured in DMEM (Gibco, USA) supplemented with 10% fetal bovine serum (Gibco, USA) and 1% penicillin-streptomycin (HyClone, USA). The 786-O cells were maintained in RPMI-1640 (Gibco, USA) with 10% fetal bovine serum and 1% penicillin-streptomycin (HyClone, USA), while ACHN and Caki-1 cells were cultured in MEM (Gibco, USA) with the same supplements. All cell cultures were maintained at 37°C in a humidified atmosphere with 5% CO_2_.

Construct lentiviral vectors targeting the down-regulation of the KIAA1429 gene. The knockdown lentivirus (LV-shKIAA1429) and its negative control (LV-shNC) were purchased from Suzhou Jima Pharmaceutical Co., Ltd. According to the manufacturer's instructions, Caki-1 and ACHN cells were infected with LV-shKIAA1429 or LV-shNC. Puromycin (3.0 μg/mL) was added to the culture medium and the cells were cultured for 14 days to screen for stable cell lines. The knockdown efficiency of KIAA1429 in stable Caki-1 and ACHN cells was verified by qRT-PCR and Western blot analysis. The interference sequences are as follows: LV-shKIAA1429-1: 5’-AGGAGTGATCAGTGGATTATT;LV-shKIAA1429-2: 5’-CCAAGAAATAGTTCGCTCTTA;LV-shNC: 5’-TTCTCCGAACGTGTCACGT.

#### Ethics approval

This study was conducted in accordance with the Declaration of Helsinki and the Ethical Guidelines for Medical and Health Research Involving Human Subjects, and was approved by the Ethics Committee of the Second Affiliated Hospital of Nantong University. (approval no. 2021KYS004). The animal experiments were approved by the Animal Ethics Committee of Nantong University (approval no. S20220227-041) and the experiments were conducted according to the National Institutes of Health Guide for the Care and Use of Laboratory Animals.

### Method details

#### Bioinformatics analysis

The GEPIA database (http://gepia.cancer-pku.cn/index.html) was used to compare KIAA1429 expression across various human malignancies and to identify differences in its expression at different clinical stages in patients with ccRCC (tumor samples n=523, normal samples n=100).^40^ Kaplan–Meier plotter survival analysis was employed to assess the correlation between KIAA1429 expression and overall survival (OS) as well as disease-free survival (DFS) in ccRCC patients. Furthermore, Gene Ontology (GO) and Kyoto Encyclopaedia of Genes and Genomes (KEGG) analyses were performed to investigate the association between KIAA1429 expression and tumour-related signalling pathways. The DESeq2 package was used to analyze the differentially expressed genes (DEGs) between the two groups (|*log*2FC|>1.5, P<0.05).

The GSE datasets (GSE36895, GSE53757, and GSE40435) related to ccRCC were retrieved from the GEO database (https://www.ncbi.nlm.nih.gov/geo/).^41^ Specifically, the GSE36895 dataset contains 23 samples of ccRCC and adjacent non-cancerous tissues,^42^ the GSE53757 dataset includes 72 such paired samples,^43^ and the GSE40435 dataset comprises 101 paired samples.^44^ To analyze the mRNA expression differences of the KIAA1429 gene between normal and tumor tissues in ccRCC patients, data from both the TCGA and GEO databases were used. The R package “limma” facilitated the differential expression analysis, while the R package “ggplot2” was employed for data visualization.

#### Western blot

Western Blot assays were performed by well-established protocols as previously described.^45^ Before the antibody hybridization, the membrane was cut according to the molecular weight of the target protein, resulting in images that were not long enough. The primary antibodies used in western blotting were as follows: KIAA1429 (1:1000, Proteintech), β-actin (1:5000, Proteintech), Vimentin (1:1000, Proteintech), N-cadherin (1:1000, Proteintech), E-cadherin (1:1000, Proteintech) and MYC (1:1000, Proteintech). The secondary antibody used in western blotting was HRP Goat Anti-Rabbit IgG (1:5000, Proteintech) and HRP Goat Anti-Mouse IgG (1:5000, Proteintech).The original WB image can be found in Table S2.

#### Immunohistochemistry (IHC) analysis

Tissue microarrays were constructed from 79 formalin-fixed and paraffin-embedded ccRCC tumour samples. IHC assays were performed as per well-established, previously described protocols.^45^ The primary antibody KIAA1429 (1:50) was added and the samples were incubated overnight at 4°C. After washing, secondary antibody HRP goat anti-rabbit IgG (1:200) was added and incubated at room temperature for 1 h. The stained tissues were graded based on staining intensity (SI) and percentage of positive cells (PP). Immunohistochemical staining was evaluated by two urologists, with low-staining (0-6) and high-staining (7-12) groups delineated according to different scores.

#### Quantitative real-time PCR

qRT-PCR assays were performed as per well-established, previously described protocols.^41^ qRT-PCR were performed using the CFX96™ Real-Time System (Bio-Rad, USA). GAPDH served as an internal control, and the 2^−ΔΔCt^ method was used for mRNA quantification. The sequences of primers are as follows:

KIAA1429-F: CCCAACGATGGCACGAATTAC.

KIAA1429-R: GGGTGAGCACTTGTTACTGGA.

MYC-F: CATCAGCACAACTACGCAGC.

MYC-R: GCTGGTGCATTTTCGGTTGT.

GAPDH-F: ACTTGGTATCGTGGAAGGACTC.

GAPDH-R: GTAGAGGCAGGGATGATGTTCTG.

#### Cell colony formation assay

Caki-1 and ACHN cells were seeded in 6-well plates at a density of 800 cells per well and cultured in MEM medium supplemented with 20% fetal bovine serum for 1 week. Cell growth was monitored, and the culture was halted once a single colony exceeded 50 cells. The colonies were then fixed with 4% paraformaldehyde for 30 minutes and stained with 0.1% crystal violet. Images of the cell colonies were subsequently captured.

#### Cell proliferation assay

CCK-8 assay was used to assess cell proliferation. Caki-1 and ACHN cells were seeded into 96-well plates at a density of 5,000 cells per well and cultured in DMEM medium supplemented with 10% fetal bovine serum for 24, 48, 72, and 96 hours. After incubation with CCK-8 for 2 hours at 37°C, the optical density (OD) was measured at 450 nm using a microplate reader.

#### Cell wound healing assay

Caki-1 and ACHN cells, either knocked down using shKIAA1429 or belonging to the control group, were seeded in 6-well plates at a density of approximately 5 × 10ˆ5 cells per well. Once the cells reached 90% confluence, the medium was replaced with serum-free culture medium. The cell layer in each well was then scratched with a pre-sterilized steel ruler and a white pipette tip. Images were captured under a microscope immediately after the scratch and subsequently every 6 hours. This process was repeated three times, and the migration rate of cells in each group was calculated.

#### Transwell assay

Caki-1 and ACHN cells (1×10^5^) with 200μl of FBS-free medium was added into the upper chamber, and 600 μl of medium with 20% FBS was add into the lower chamber. After incubating for 24 hours at 37°C with 5% CO2, non-invasive cells were removed on the upper surface by a cotton swab. The invaded cells were fixed with 4% paraformaldehyde, stained with 0.1% crystal violet and counted with the microscope.

#### Cell cycle assay

Caki-1 and ACHN cells (1×10^6^) were harvested, washed with pre-chilled PBS, fixed in 75% pre-chilled ethanol at 4°C and incubated overnight. The cells were stained using propidium iodide (BD, USA) at 15°C for 30 min. Flow cytometry was then employed (Becton Dickinson, USA), with Cell Quest Modfit software facilitating cell cycle analysis.

#### mRNA stability assay

Caki-1 and ACHN cells were seeded into 6-well plates (5×10^5^ cells/well) and incubated at 37°C with 5% CO2. Subsequently, ccRCC cells were treated with Actinomycin D (5 μg/mL) for 0, 2, 4 and 6 h. Relative MYC mRNA expression was detected using qRT-PCR and normalized to the 0 h group.

#### RNA immunoprecipitation (RIP)

Magna RIPTM RNA-binding Protein Immunoprecipitation Kit (Millipore, USA) facilitated RIP experiments. Briefly, Caki-1 and ACHN cells (2×107 ) were lysed with RIP lysis buffer, co-incubated with 10 μg of magnetic beads conjugated with antiKIAA1429 (Proteintech, USA) and non-immunized with IgG overnight at 4°C. After RNA was purified, qRT-PCR was performed to assess MYC transcription within KIAA1429 or IgG immunocomplexes.

#### m6A RNA methylation detection

Total cellular RNA was extracted as described above, and genomic DNA was removed using gDNA wiper. The global m6A methylation level in mRNA was quantified using the EpiQuik m6A RNA Methylation Quantification Kit (Merck Millipore, Germany) according to the manufacturer's protocol. Absorbance was measured at 450 nm using a microplate reader, with 200 ng of mRNA used for each sample analysis.

#### MeRIP-qPCR assay

Magna MeRIP™ m6A Kit (Merck Millipore, Germany) measured m6A levels in MYC mRNA. Briefly, 100 μg of total RNA was treated with gDNA wiper mix (Vazyme, China) and the concentration was adjusted to 1 μg/μl with RNAase-free water. Following this, chemically fragmented RNA (100-nt) was immunoprecipitated with an anti-m6A antibody (Synaptic, Germany) according to the manufacturer’s instructions, and 1/10 of the fragmented RNA was saved as input control. Subsequent qRT-PCR compared m6A enrichment, normalized to input.

#### Luciferase assay

The wild-type reporter plasmid pmirGLO-MYC-WT was constructed by inserting the 3′ UTR of MYC downstream of the firefly luciferase (Fluc) coding sequence. To generate the mutant reporter pmirGLO-MYC-MUT, the adenine within the predicted m6A consensus sequence was substituted with cytosine. Both plasmids were obtained from ProMega Corporation (Milwaukee, USA).

#### Statistical analysis

Statistical analysis was performed using GraphPad 8.02 software. All experiments were repeated three times. Data are presented as mean ± standard deviation (SD). Chi-square test and Student’s t test were used to determine differences among two groups. The analysis of differences among multiple groups was evaluated through one-way ANOVA analysis and Tukey’s test. Survival curves were drawn using the Kaplan-Meier method and compared by log-rank test. P value < 0.05 was considered statistically significant.

## Supplemental information

Data S1. The detailed personal information of each participant
